# Informative gene selection and the direct classification of tumors based on relative simplicity

**DOI:** 10.1186/s12859-016-0893-0

**Published:** 2016-01-20

**Authors:** Yuan Chen, Lifeng Wang, Lanzhi Li, Hongyan Zhang, Zheming Yuan

**Affiliations:** Hunan Provincial Key Laboratory for Biology and Control of Plant Diseases and Insect Pests, Changsha, China; Hunan Provincial Key Laboratory for Germplasm Innovation and Utilization of Crop, Hunan Agricultural University, Changsha, China; Biotechnology Research Center, Hunan Academy of Agricultural Sciences, Changsha, China

**Keywords:** Microarray expression data, Gene selection, Direct classify, Relative simplicity, Binary-discriminative informative genes, Paired votes

## Abstract

**Background:**

Selecting a parsimonious set of informative genes to build highly generalized performance classifier is the most important task for the analysis of tumor microarray expression data. Many existing gene pair evaluation methods cannot highlight diverse patterns of gene pairs only used one strategy of vertical comparison and horizontal comparison, while individual-gene-ranking method ignores redundancy and synergy among genes.

**Results:**

Here we proposed a novel score measure named relative simplicity (RS). We evaluated gene pairs according to integrating vertical comparison with horizontal comparison, finally built RS-based direct classifier (RS-based DC) based on a set of informative genes capable of binary discrimination with a paired votes strategy. Nine multi-class gene expression datasets involving human cancers were used to validate the performance of new method. Compared with the nine reference models, RS-based DC received the highest average independent test accuracy (91.40 %), the best generalization performance and the smallest informative average gene number (20.56). Compared with the four reference feature selection methods, RS also received the highest average test accuracy in three classifiers (Naïve Bayes, k-Nearest Neighbor and Support Vector Machine), and only RS can improve the performance of SVM.

**Conclusions:**

Diverse patterns of gene pairs could be highlighted more fully while integrating vertical comparison with horizontal comparison strategy. DC core classifier can effectively control over-fitting. RS-based feature selection method combined with DC classifier can lead to more robust selection of informative genes and classification accuracy.

**Electronic supplementary material:**

The online version of this article (doi:10.1186/s12859-016-0893-0) contains supplementary material, which is available to authorized users.

## Background

Microarray expression data of cancer tissue samples has the following properties: small sample size yet large number of features, high noise and redundancy, a remarkable level of background differences among samples and features, and nonlinearity [1, 2]. Selecting a parsimonious set of informative genes to build robust classifier with highly generalized performance is one of the most important tasks for the analysis of microarray expression data, as it can help to discover disease mechanisms, as well as improve the precision and reduce the cost of clinical diagnoses [3].

Gene selection depends on a given evaluation strategy and a defined score. The individual-gene-ranking methods rank genes by only comparing the expression values of the same individual gene between different classes (a vertical comparison evaluation strategy). This can be very far from the truth, as the deregulation of pathways, rather than individual genes, may be critical in triggering carcinogenesis [4]. If a gene has a remarkable joint effect on other genes, it should be selected as an informative gene, even though it may receive a lower rank in an individual-gene-ranking method. This joint effect of genes has been taken into account in most popular, existing algorithms, including top scoring pair (TSP) [5, 6], top scoring triplet(TST) [7], top-scoring ‘N’(TSN) [8], top scoring genes (TSG) [9] and doublet method [4]. However, the gene pairs score, that is the percentage of Δ_*ij*_ in TSP [5, 6], cannot reflect size differences among samples. To fully utilize sample size information TSG introduces chi-square values as the score for gene pairs [9]. TSP and TSG are both pair-wise gene evaluations, which compare the expression values of the same sample between two different genes (a horizontal comparison evaluation strategy), and can help to eliminate the influence of sampling variability due to different subjects [5, 6, 9].

At the level of gene pairs, Merja *et al.* [10] defined two patterns based on rank data, rather than absolute expression, from data-driven perspective: the consistent reversal of relative expression and consistent relative expression. This premise allowed us to organize the cell types in to their ontogenetic lineage-relationships and may reflect regulatory relationships among the genes [10]. The first pattern can be subdivided into a consistent reversal of expression (Pattern I) and a consistent reversal of relative expression (Pattern II) based on absolute expression (see Table 1). Similarly, the second pattern can be subdivided in to a consistent expression (Pattern III) and a consistent relative expression (Pattern IV). Furthermore, a heterogeneous background expression of samples (Pattern V) and an interaction expression pattern (Pattern VI) can be defined, if the influence of sampling variability due to different subjects [9] and paired-gene interactions are considering [11]. Clearly, all twelve genes (G_1_ ~ G_12_) in Table 1 should be informative genes from data-driven perspective. However, individual-gene evaluations, which only detect different expression levels between positive samples and negative samples, cannot highlight Pattern V and Pattern VI. Pair-wise gene evaluation with vertical comparison can highlight most patterns except Pattern V. Only pair-wise gene evaluation with horizontal comparison can highlight Pattern V, even though it cannot detect most other patterns. Therefore, both vertical and horizontal comparisons need to be considered in pair-wise gene evaluation techniques.

**Table 1:** Six patterns for joint effect of gene pairs in binary-class simulation data

Class		Pattern I				Pattern II				Pattern III				Pattern IV				Pattern V				Pattern VI			
		G1		G2		G3		G4		G5		G6		G7		G8		G9		G10		G11		G12	
+		50		100		5		100		50		50		5		50		50		100		50		50	
+		50		100		5		100		50		50		5		50		5		10		100		100	
+		50		100		5		100		50		50		5		50		50		100		50		50	
+		50		100		5		100		50		50		5		50		5		10		100		100	
-		100		50		10		50		100		100		10		100		100		50		50		100	
-		100		50		10		50		100		100		10		100		10		5		100		50	
-		100		50		10		50		100		100		10		100		100		50		50		100	
-		100		50		10		50		100		100		10		100		10		5		100		50	
Background difference between gene pairs		Not exist				Exist				Not exist				Exist				Not exist				Not exist			
Background difference among samples		Not exist				Not exist				Not exist				Not exist				Exist				Not exist			
Vertical comparison of individual-gene		G1 < 75	G1 > 75	G2 < 75	G2 > 75	G3 < 7	G3 > 7	G4 < 75	G4 > 75	G5 < 75	G5 > 75	G6 < 75	G6 > 75	G7 < 7	G7 > 7	G8 < 75	G8 > 75	G9 < 41	G9 > 41	G10 < 41	G10 > 41	G11 < 75	G11 > 75	G12 < 75	G12 > 75
	+	4	0	0	4	4	0	0	04	4	0	4	0	4	0	4	0	2	2	2	2	2	2	2	2
	-	0	4	4	0	0	4	4	0	0	4	0	4	0	4	0	4	2	2	2	2	2	2	2	2
Highlight		Yes (*χ* ^2^ = 4.5^*^)		Yes (*χ* ^2^ = 4.5^*^)		Yes (*χ* ^2^ = 4.5^*^)		Yes (*χ* ^2^ = 4.5^*^)		Yes (*χ* ^2^ = 4.5^*^)		Yes (*χ* ^2^ = 4.5^*^)		Yes (*χ* ^2^ = 4.5^*^)		Yes (*χ* ^2^ = 4.5^*^)		No (*χ* ^2^ = 0.5)		No (*χ* ^2^ = 0.5)		No (*χ* ^2^ = 0.5)		No (*χ* ^2^ = 0.5)	
Horizontal comparison of pair-wise genes		G1 > G2		G1 < G2		G3 > G4		G3 < G4		G5 > G6		G5 < G6		G7 > G8		G7 < G8		G9 > G10		G9 < G10		G11 > G12		G11 < G12	
	+	0		4		0		4		2		2		0		4		0		4		2		2	
	-	4		0		0		4		2		2		0		4		4		0		2		2	
Highlight		Yes (*χ* ^2^ = 4.5^*^)				No (*χ* ^2^ = 0)				No (*χ* ^2^ = 0.5)				No (*χ* ^2^ = 0)				Yes (*χ* ^2^ = 4.5^*^)				No (*χ* ^2^ = 0.5)			
Vertical comparison of pair-wise genes		G1 < 75 & G2 < 75	G1 < 75 &G2 > 75	G1 > 75 & G2 < 75	G1 > 75 &G2 > 75	G3 < 7 & G4 < 75	G3 < 7 & G4 > 75	G3 > 7 & G4 < 75	G3 > 7 & G4 > 75	G5 < 75 & G6 < 75	G5 < 75 & G6 > 75	G5 > 75 & G6 < 75	G5 > 75 & G6 > 75	G7 < 7 & G8 < 75	G7 < 7 & G8 > 75	G7 > 7 & G8 < 75	G7 > 7 & G8 > 75	G9 < 41 & G10 < 41	G9 < 41 & G10 > 41	G9 > 41 & G10 < 41	G9 > 41 & G10 > 41	G11 < 75 & G12 < 75	G11 < 75 & G12 > 75	G11 > 75 & G12 < 75	G11 > 75 & G12 > 75
	+	0	4	0	0	0	4	0	0	4	0	0	0	0	4	0	0	2	0	0	2	2	0	0	2
	-	0	0	4	0	0	0	4	0	0	0	0	4	0	0	0	4	2	0	0	2	0	2	2	0
Highlight		Yes (*χ* ^2^ = 4.5^*^)				Yes (*χ* ^2^ = 4.5^*^)				Yes (*χ* ^2^ = 4.5^*^)				Yes (*χ* ^2^ = 4.5^*^)				No (*χ* ^2^ = 0.5)				Yes ((*χ* ^2^ = 8^*^)			

Values in parenthesis are chi-square values, * denote *p* < 0.05

We first propose a novel score measure, in this paper, that of relative simplicity (RS), based on information theory. We adopt an integrated evaluation strategy to rank genes one by one, considering not only individual-gene effects, but also pair-wise joint effects between candidate gene and others. In particular, for pair-wise gene evaluations, vertical comparisons are integrated with horizontal comparisons to detect all six patterns of pair-wise joint effects. Ultimately, we construct a relative simplicity-based direct classifier (RS-based DC) to select binary-discriminative informative genes on training dataset and perform independent tests. The independent testing of nine multiclass tumor gene expression datasets showed that RS-based DC selects fewer informative genes and outperforms the referred models by a large margin, especially in larger *m* (total number of classes) datasets, such as Cancers (*m* = 11) [12]and GCM (*m* = 14) [13].

## Datasets and methods

### Datasets

Ten multi-class datasets have been used in published previous TSP [5, 6] and TSG [9] papers. We did not include dataset Leukemia3 [14] in our study because 65 % of the expression values in it are zero. The remaining nine datasets references, sample sizes, numbers of genes, and numbers of classes are summarized in Table 2. Suppose that a training dataset has *n* samples and *p* genes, and that the data can be denoted as (*Y*_*i*_, *X*_*i,j*_), *i* = 1,2,…, *n*; *j* = 1,2,…, *p*. Where *X*_*i,j*_ represents the expression value of the *j*^th^ gene (G_*j*_) in the *i*^th^ sample; and *Y*_*i*_ represents the class label of *i*^th^ sample, where *Y*_*i*_∈{Class_1_, Class_2_, …, Class_*t*_, …, Class_*m*_}, *t* = 1,2,…,*m*.

**Table 2:** Nine multi-class gene expression datasets

Dataset	Platform	No. of classes	No. of genes	No. of samples		Source
				Training	Test	
Leukemia1	Affy	3	7129	38	34	[15]
Lung1	Affy	3	7129	64	32	[16]
Leukemia2	Affy	3	12582	57	15	[17]
SRBCT	cDNA	4	2308	63	20	[18]
Breast	Affy	5	9216	54	30	[19]
Lung2	Affy	5	12600	136	67	[20]
DLBCL	cDNA	6	4026	58	30	[21]
Cancers	Affy	11	12533	100	74	[12]
GCM	Affy	14	16063	144	46	[13]

## Data preprocessing

### Adjustment for outliers

Outliers may exist in datasets. For example, in the Lung1 [16] training set, the expression value *X*_54,4290_ of the 54^th^ sample in gene G_4290_ is 7396.1, while the average expression value of the other samples in gene G_4290_ is 80.15 (range from 16 to 197). The outliers overstate the differences among the classes, and need be adjusted before gene ranking. For gene G_*j*_, we defined outliers as those values beyond the scope of [$\overline{X}{.}_j-{u}_{\alpha}\sigma {.}_j$, $\overline{X}{.}_j+{u}_{\alpha}\sigma {.}_j$]. If ${X}_{ij}<\overline{X}{.}_j-{u}_{\alpha}\sigma {.}_j$ or ${X}_{ij}>\overline{X}{.}_j-{u}_{\alpha}\sigma {.}_j$, then *X*_*ij*_ is an outlier, where α is significance level, $\overline{X}{.}_j$ and *σ*. _*j*_ represent the average value and standard deviation of *X* · *j*, respectively. Therefore, we adjust the outliers using the following formula:

1 $$ {X}_{ij}^{"}=\left\{\begin{array}{l}{\overline{X}}_{\hbox{-} i,j}-u\alpha {\sigma}_{\hbox{-} i,j}\kern1.25em \mathrm{if}\kern0.5em {X}_{ij} < \overline{X}{.}_j-u\alpha \sigma {.}_j\\ {}\\ {}{\overline{X}}_{\hbox{-} i,j}+u\alpha {\sigma}_{\hbox{-} i,j}\kern1.25em \mathrm{if}\kern0.5em {X}_{ij} > \overline{X}{.}_j+u\alpha \sigma {.}_j\end{array}\right. $$

Here ${\overline{X}}_{\hbox{-} i,j}$ and *σ*_‐ *i*,*j*_ represent the average value and standard deviation of *X* · *j* without *X*_*i,j*_, respectively. *X*_*ij*_^"^ is the value of *X*_*ij*_ after adjusting. $\left[{\overline{X}}_{\hbox{-} i,j}-{u}_{\alpha }{\sigma}_{\hbox{-} i,j},{\overline{X}}_{\hbox{-} i,j}+{u}_{\alpha }{\sigma}_{\hbox{-} i,j}\right]$ represents the distribution interval of *X*_*-i,j*_. We generally set α to 0.05 (*u*0.05 = 1.96). Adjustment for outliers was only used with training set.

### Transforming datasets from multi-class to binary-class with “one versus rest”

Suppose that *Y*_*i*_∈(Class_1_, Class_2_, …, Class_*t*_, …, Class_*m*_), and we adopt a “one versus rest” (OVR) approach to transform a multi-class training set to binary-class. This generates *m* binary-class datasets, denoted {Class_1_*vs.* non-Class_1_}, {Class_2_*vs.* non-Class_2_}, …, {Class_*t*_*vs.* non-Class_*t*_}, …, {Class_*m*_*vs.* non-Class_*m*_}. In each binary-class training dataset, Class_*t*_ are positive samples {+}, and non-Class_*t*_ are negative samples {−}.

## Complexity and relative simplicity score

Entropy stands for disorder or uncertainty. For a discrete system with *k* events, its Shannon entropy is defined as:

2 $$ H=-{\displaystyle \sum_{i=1}^k\frac{n_i}{N} \log \left(\frac{n_i}{N}\right)} $$

Where *n*_*i*_ denotes the frequency of event *i*, and *N* is the total frequency. Here we use base-2 logarithms. *H* only reflects the event ratios. Complexity (*C*) as proposed by Zhang [22] can reflect both event ratios and event frequencies:

3 $$ C=-{\displaystyle \sum_{i=1}^k{n}_i \log \left(\frac{n_i}{N}\right)} $$

For a given 2 × *r* Contingency table (Table 3), its complexity is the total of row complexities (*C*_row_) and column complexities (*C*_column_). f_+*d*_ (*d* = 1,…,*r*) and f_−*d*_ in Table 3 represent the frequency of the event.

4 $$ {C}_{\mathrm{row}}=-{\displaystyle \sum_{d=1}^r{\mathrm{f}}_{+d} \log \left(\frac{{\mathrm{f}}_{+d}}{{\mathrm{f}}_{+}}\right)} - {\displaystyle \sum_{d=1}^r{\mathrm{f}}_{-d} \log \left(\frac{{\mathrm{f}}_{-d}}{{\mathrm{f}}_{-}}\right)} $$

5 $$ {C}_{\mathrm{column}}=-{\displaystyle \sum_{d=1}^r\Big({\mathrm{f}}_{+d} \log \left(\frac{{\mathrm{f}}_{+d}}{{\mathrm{f}}_d}\right)+}{\mathrm{f}}_{-d} \log \left(\frac{{\mathrm{f}}_{-d}}{{\mathrm{f}}_d}\right)\Big) $$

6 $$ C={C}_{\mathrm{row}}+{C}_{\mathrm{column}} $$

**Table 3:** 2×*r* Contingency table

Class	Column_1_	…	Column_*d*_	…	Column_*r*_	Total
+	f_+1_	…	f_+*d*_	…	f_+*r*_	f_+_
-	f_-1_	…	f_-*d*_	…	f_-*r*_	f_-_
Total	f_1_	…	f_*d*_	…	f_*r*_	*n*

For contingency Table 1 (2 × *r*_1_) and contingency Table 2 (2 × *r*_2_), their complexities are incomparable if *r*_1_ is unequal to *r*_2_. Therefore we introduce a novel score, RS, according to their maximum complexity (Table 4). Table 4 cames directly from Table 3 directly, only the frequency of each column in the same class is set to be equal.

7 $$ {C}_{\mathrm{row}\hbox{-} \max }=n \log (r) $$

8 $$ {C}_{\mathrm{column}\hbox{-} \max }=-{\mathrm{f}}_{+} \log \left(\frac{{\mathrm{f}}_{+}}{n}\right)-{\mathrm{f}}_{-} \log \left(\frac{{\mathrm{f}}_{-}}{n}\right) $$

9 $$ {C}_{\max }={C}_{\mathrm{row}\hbox{-} \max }+{C}_{\mathrm{column}\hbox{-} \max } $$

10 $$ RS=\frac{C_{\max }-C}{C_{\max }} $$

**Table 4:** 2×*r* Contingency table for maximum complexity

Class	Column_1_	…	Column_*d*_	…	Column_*r*_	Total
+	f_+_/*r*	…	f_+_/*r*	…	f_+_/*r*	f_+_
-	f_-_/*r*	…	f_-_/*r*	…	f_-_/*r*	f_-_
Total	*n*/*r*	…	*n*/*r*	…	*n*/*r*	*n*

## Individual-gene evaluation

For a given gene G_*j*_ with continued expression values *X*. _*j*_ in a binary-class training dataset, we partition *X*. _*j*_ into two parts (*X*. _*j*_ > *EP*_*j*_ and *X*. _*j*_ < *EP*_*j*_) with an endpoint (*EP*):

11 $$ E{P}_j=\left({\overline{X}}_{-j}+{\overline{X}}_{+j}\right)/2 $$

Where ${\overline{X}}_{-j}$ and ${\overline{X}}_{+j}$ are the average expression values of *X*. _*j*_ for negative and positive samples, respectively. We then generate a 2 × 2 contingency table for gene *G*_*j*_ (Table 5).

**Table 5:** 2 × 2 contingency table for individual gene

Class	*X*. _*j*_ > *EP*_*j*_	*X*. _*j*_ < *EP*_*j*_	Total
+	f_+1_	f_+2_	f_+_
-	f_−1_	f_−2_	f_−_
Total	f_1_	f_2_	*n*

f_+1_ is the number of positive samples with expression values larger than *EP*_*j*_, f_+2_ is the number of positive samples with expression values less than *EP*_*j*_, f_−1_ is the number of negative samples with expression values larger than *EP*_*j*_, and f_−2_ is the number of negative samples with expression values less than *EP*_*j*_. When *X*_*i,j*_ equals *EP*_*j*_, and *Y*_*i*_ belongs to positive sample {+}, f_+1_ and f_+2_ increase by 0.5 respectively; when *X*_*i,j*_ equals *EP*_*j*_, and *Y*_*i*_ belongs to negative sample {−}, f_−1_ and f_−2_ increase by 0.5 respectively

For the individual-gene evaluation of gene G_*j*_, we then got its RS score, $R{S}_{G_j}$, according to Table 5 and formula (10).

## Pair-wise gene evaluation

### Horizontal comparison of gene pairs

For gene pairs *G*_*j*_ and *G*_*q*_ (*j ≠ q*) in a binary-class training dataset, we generate a 2 × 2 contingency table (Table 6) for the horizontal comparison with *X*_*i,j*_ > *X*_*i,q*_ and *X*_*i,j*_ < *X*_*i,q*_, similar to TSP [2, 3] and TSG [9].

**Table 6:** 2 × 2 contingency table for gene pairs of horizontal comparison

Class	*X*_*i,j*_ > *X*_*i,q*_	*X*_*i,j*_ < *X*_*i,q*_	Total
+	f_+1_	f_+2_	f_+_
-	f_−1_	f_−2_	f_−_
Total	f_1_	f_2_	*n*

*X*_*i,j*_ represents the expression value of the *j* ^th^ gene (G_*j*_) in the *i* ^th^ sample; f_+1_ is the number of positive samples with *X*_*i,j*_ larger than *X*_*i,q*_, f_+2_ is the number of positive samples with *X*_*i,j*_ less than *X*_*i,q*_, f_−1_ is the number of negative samples with *X*_*i,j*_ larger than *X*_*i,q*_, and f_−2_ is the number of negative samples with *X*_*i,j*_ less than *X*_*i,q*_

For horizontal comparison of gene pairs *G*_*j*_ and *G*_*q*_, We generate the complexity *C*_*hor-Gj-Gq*_ and the maximum complexity *C*_*hor-Gj-Gq-*max_, of gene pairs *G*_*j*_ and *G*_*q*_, for the horizontal comparison, according to Table 6, formula (6), and formula (9).

### Vertical comparison of gene pairs

For gene pairs *G*_*j*_ and *G*_*q*_ (*j* ≠ *q*) in a binary-class training dataset, we partition *X*. _*j*_ and *X*. _*q*_ into two parts with endpoint *EP*_*j*_ and *EP*_*q*_, respectively. We then generate a 2 × 4 contingency table (Table 7) for the vertical comparison.

**Table 7:** 2 × 4 contingency table for gene pairs of vertical comparison

Class	*X*. _*j*_ > *EP*_*j*_ & *X*. _*q*_ > *EP*_*q*_	*X*. _*j*_ > *EP*_*j*_ & *X*. _*q*_ < *EP*_*q*_	*X*. _*j*_ < *EP*_*j*_ & *X*. _*q*_ > *EP*_*q*_	*X*. _*j*_ < *EP*_*j*_ & *X*. _*q*_ < *EP*_*q*_	Total
+	f_+1_	f_+2_	f_+3_	f_+4_	f_+_
-	f_−1_	f_−2_	f_−3_	f_−4_	f_−_
Total	f_1_	f_2_	f_3_	f_4_	*n*

f_+1_ is the number of positive samples with *X.*_*j*_ larger than *EP*_*j*_ and *X.*_*q*_larger than *EP*_*q*_, f_+2_ is the number of positive samples with *X.*_*j*_ larger than *EP*_*j*_ and *X.*_*q*_ less than *EP*_*q*_, f_+3_ is the number of positive samples with *X.*_*j*_ less than *EP*_*j*_ and *X.*_*q*_ larger than *EP*_*q*_, f_+4_ is the number of positive samples with *X.*_*j*_ less than *EP*_*j*_ and *X.*_*q*_ less than *EP*_*q*_, f_−1_ is the number of positive samples with *X.*_*j*_ larger than *EP*_*j*_ and *X.*_*q*_ larger than *EP*_*q*_, f_−2_ is the number of positive samples with *X.*_*j*_ larger than *EP*_*j*_ and *X.*_*q*_ less than *EP*_*q*_, f_−3_ is the number of positive samples with *X.*_*j*_ less than *EP*_*j*_ and *X.*_*q*_ larger than *EP*_*q*_, and f_−4_ is the number of positive samples with *X.*_*j*_ less than *EP*_*j*_ and *X.*_*q*_ less than *EP*_*q*_

For vertical comparison of gene pairs *G*_*j*_ and *G*_*q*_, We then generate the complexity *C*_*ver-Gj-Gq*_ and the maximum complexity *C*_*ver -Gj-Gq-*max_ of gene pairs *G*_*j*_ and *G*_*q*_ for the vertical comparison according to Table 7, formula (6), and formula (9).

#### RS score of gene pairs

For gene pairs *G*_*j*_ and *G*_*q*_ in a binary-class training dataset, we generate RS weight scores, *RS*_*Gj*_*Gq*_, according to formula (12).

12 $$ R{S}_{Gj\_Gq}=\frac{\left({C}_{hor-Gj-Gq- \max }+{C}_{ver-Gj-Gq- \max}\left)-\right({C}_{hor-Gj-Gq}+{C}_{ver-Gj-Gq}\right)}{C_{hor-Gj-Gq- \max }+{C}_{ver-Gj-Gq- \max }} $$

## Integrated individual-gene ranking

For a given gene G_*j*_ in a binary-class training dataset, the integrated RS score, *IRS*_*Gj*_, can be calculated with formula (13):

13 $$ IR{S}_{Gj}=R{S}_{Gj}+{\displaystyle \sum_{q=1}^p\left(\frac{R{S}_{Gj}}{R{S}_{Gj}+R{S}_{Gq}}\times R{S}_{Gj\_Gq}\right)},q\ne j $$

**Fig. 1:**
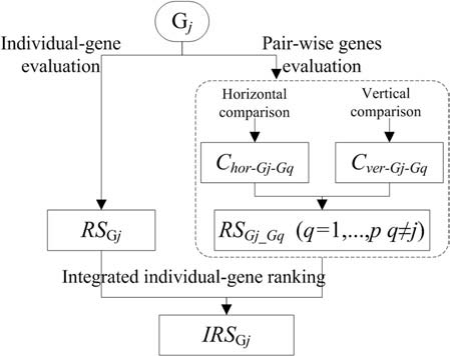
Integrated evaluation process of G_*j*_

Here, *RS*_*Gj*_ represents vertical comparison of individual-gene; *RS*_*Gj_Gq*_ represents horizontal comparison and vertical comparison of pair-wise genes; $\frac{R{S}_{Gj}}{R{S}_{Gj}+R{S}_{Gq}}$ represents the weight of G*j* in the pair-wise comparison. According to *IRS*_*Gj*_, the descending order of all *p* genes can be obtained and recorded as {G_Rank1_, G_Rank2_,…, G_Rank*j*_,…, G_Rank*p*_}. The integrated evaluation process of G_*j*_ is shown in Fig. 1.

## Informative gene selection

The *IRS* scores provide a list of top ranked genes. However, the combination of top ranked genes may not produce a top ranked combination of genes because of the redundancy and interaction among genes [23]. Therefore, we used a forward feature selection strategy to select informative gene subsets, along with our RS-based-DC classifier and leave-one-out cross-validation error estimates (LOOCV).

For a given binary-class training dataset with *n* samples and *p* ranked genes:

Step 1: Introduce gene G_Rank1_, get dataset *S*∈(*Y*_*i*_, *X*_*i*_), *i* = 1,2,…, *n*; *X*_*i*_ represents the expression value of gene G_Rank1_ in the *i*^th^ sample; *Y*_*i*_ represents the class label of *i*^th^ sample and *Y*_*i*_∈{+, −}. Leave out one sample as the validation data (*S*-validation) and the rest as the training data (*S*-train). First assign {+} to S-validation as a class label, merge S-validation and S-train, get *RS*_GRank1_(+); then assign {−} to *S*-validation as a class label, merge *S*-validation and *S*-train, get *RS*_GRank1_(−). If *RS*_GRank1_(+) is larger than *RS*_GRank1_(−), the *S*-validation sample belongs to the positive sample; otherwise, the *S*-validation sample belongs to the negative sample. Repeat prediction for all the samples in *S* to get the prediction class labels. Calculate the Matthew correlation coefficient (MCC) according to formula (14) and denote as *MCC*_1_.

14 $$ MCC=\frac{\left(TP\times TN\right)-\left(FN\times FP\right)}{\sqrt{\left(TP+FN\right)\times \left(TN+FP\right)\times \left(TP+FP\right)\times \left(TN+FN\right)}} $$

Here *TP*, *TN*, *FP*, *FN* represent true positives, true negatives, false positives and false negatives, respectively.

Step 2: MCC_benchmark_ = *MCC*_1_.

Step 3: Introduce the next top ranked gene. In general, denote total number of the current genes as *r*. Get dataset *S* = (*Y*_*i*_, *X*_*i,j*_), *i* = 1,2,…, *n*; *j* = 1,2,…, *r*. The network RS score of *r* gene can be calculated with formula (15).

15 $$ R{S}_r\hbox{-} net={\displaystyle \sum_{j=1}^r{\displaystyle \sum_{q=1}^rR{S}_{GRankj\_ GRankq}}},q\ne j $$

Leave out one sample as the validation data (*S*-validation) and the rest as the training data (*S*-train). First assign {+} to *S*-validation as a class label, merge *S*-validation and *S*-train, get *RS*_*r*_-*net*(+); then assign {−} to *S*-validation as a class label, merge *S*-validation and *S*-train, get *RS*_*r*_-*net* (−). If *RS*_*r*_-*net* (+) is larger than *RS*_*r*_-*net* (−), the *S*-validation sample belongs to the positive sample; if *RS*_*r*_-*net* (+) is less than *RS*_*r*_-*net* (−), the *S*-validation sample belongs to the negative sample. Repeat prediction for all the samples in *S* to get the prediction class labels. Calculate MCC according to formula (14) and denote as *MCC*_*r*_.

Step 4: If *MCC*_*r*_ ≤ MCC_benchmark_ delete *X*. _*r*_, else MCC_benchmark_ = *MCC*_*r*_.

Step 5: Repeat Step 3 and Step 4, until the top *B* rank genes are successively introduced (our experience suggests that it is sufficient to set the upper bound of *B* at 100).

We consequently generate the informative genes subset for the binary-class dataset (Pseudo-code see Table 8).

**Table 8:** Pseudo-code of informative genes selection

Algorithm 1 Informative gene selection (Dateset, G_Rank_)
Require: Dateset is a binary-class training dataset with *n* samples
Require: G_Rank_ is the order of all *p* genes {G_Rank1_, G_Rank2_,…, G_Rank*j*_,…, G_Rank*p*_}
Ensure: Returns the binary-discriminative informative genes subset of Dateset
1: ture_*Y* ← class lable of training samples
2: *j* ← 1; MCC_benchmark_ ← 0; *B* ← 100
3: repeat
4: *S* ← G_Rank*j*_ # introducing G_Rank*j*_
5: if |*S*| ≤ 1 then
6: for *i* = 1 to *n* do # leave-one-out cross-validation
7: *Y*_*i*_ ← +
8: get *RS*_GRank*j*_(+)
9: *Y*_*i*_ ← −
10: get *RS*_GRank*j*_(−)
11: if *RS*_GRank*j*_(+) > *RS*_GRank*j*_(−) then pred_*Y*_*i*_ ← +
12: else pred_*Y*_*i*_ ← −
13: end for
14: MCC_benchmark_ ← get MCC (true_*Y*, pred_*Y*) from formula (14)
15: else
16: for *i* = 1 to *n* do # leave-one-out cross-validation
17: *Y*_*i*_ ← +
18: get *RS*-*net*(+) from formula (15)
19: *Y*_*i*_ ← −
20: get *RS*-*net*(−) from formula (15)
21: if *RS*-*net*(+) > *RS*-*net* (−) then pred_*Y*_*i*_ ← +
22: else pred_*Y*_*i*_ ← −
23: end for
24: MCC ← get MCC (true_*Y*, pred_*Y*) from formula (14)
25: end if
26: if MCC > MCC_benchmark_ then MCC_benchmark_ ← MCC
27: else delete G_Rank*j*_
28: until *j* > B
29: retrun *S*

### Paired votes prediction with RS-based DC

We generate an *m* binary-class training set, denoted as {Class_1_*vs.* non-Class_1_}, {Class_2_*vs.* non-Class_2_},…,{Class_*t*_*vs.* non-Class_*t*_},…,{Class_*m*_*vs.* non-Class_*m*_}, according our OVR approach; and the corresponding *m* binary-discriminative informative gene (BDIG) subsets, denoted as BDIG_Class1_, BDIG_Class2_, …, BDIG_Class*t*_, …, BDIG_Class*m*_, according to our *individual-gene evaluation ~ informative gene selection* sections.

For a test sample with *m* possible class labels, in general, for paired vote predictions between Class_*t*_ and Class_*w*_, we merge the Class_*t*_ and Class_*w*_ samples into a new training set with *r* genes according to {BDIG_Class*t*_ ∪ BDIG_Class*w*_}. We first assign {Class_*t*_} to the test sample as a class label, merge the test sample and the new training set, generating *RS*_*r*_ ‐ *net* {Class_*t*_}; then we assign {Class_*w*_} to the test sample as class a label, merge the test sample and the new training set, generating *RS*_*r*_ ‐ *net* {Class_*w*_}. If *RS*_*r*_-*net* {Class_*t*_} is larger than *RS*_*r*_-*net* {Class_*w*_}, the test sample belongs to Class_*t*_, else it belongs to Class_*w*_. The winner continues paired vote with the next class and the prediction class label of the test sample is the last winner.

After the predictions for all of the testing samples have been obtained, we calculate the test accuracy, expressed as the ratio of the number of correctly classified samples to the total number of samples, for multi-classification.

## Results and analysis

### Comparison of independent prediction accuracy and the number of informative genes among different models

We used nine reference models, HC-TSP [3], HC-K-TSP [3], DT [24], PAM [25], TSG [9], mRMR-SVM, SVM-RFE-SVM, Entropy-based DC and *χ*^2^-based DC, to evaluate the performance of RS-based DC. Results from the first five models are cited from the corresponding literature, and the results from the latter four models are presented in this paper.

As a feature selection method mRMR has two evaluation criterions: mutual information difference (MID) and mutual information quotient (MIQ). Here we used MIQ-mRMR, because MIQ is more robust than MID in general [26]. mRMR and SVM-RFE [27] only provide a list of ranked genes, therefore, we adopted the Library for Support Vector Machines (LIBSVM) as a classifier [28] to generate an informative gene subset. LIBSVM supports multiclass classification, and is available at http://www.csie.ntu.edu.tw/~cjlin/libsvm. We initially listed the top 2 % of informative genes according to mRMR or SVM-RFE. Second, we introduced these genes one by one and conducted 10-fold cross-validation for the training sets based on SVM. Third, we selected the genes with the highest cross-validation accuracy as our informative genes subset, and finally we performed independent predictions using SVM with informative genes, for the mRMR-SVM and SVM-RFE-SVM models. Four kernel functions, linear, radius basis function (RBF), sigmoid and polynomial in SVM, were evaluated, and the linear kernel produced optimal accuracy with the nine datasets. Therefore, we used linear kernel in this study, unless specifically stated. Different penalty parameters *C* (*C*∈[2^−5^, 2^15^]) were optimized in different SVM models with the training set. Entropy-based DC and *χ*^2^-based DC uses the same modelling process as RS-based DC, except entropy [29] is used, rather than complexity, in Entropy-based DC, and *χ*^2^ is used, rather than RS, in *χ*^2^-based DC.

The test accuracy and informative gene number for nine different multi-class datasets are listed in Table 9. The best models based on average accuracy were RS-based DC (91.40 %), *χ*^2^-based DC (89.41 %), TSG (88.99 %), PAM (87.91 %), SVM-RFE-SVM (86.23 %) and HC-K-TSP (85.45 %). Of the six models, *χ*^2^-based DC, TSG and HC-K-TSP performed poorly in predictive power with GCM, Cancers and Breast datasets, respectively. PAM generated an unacceptable informative gene number (an average of 1450), and also demonstrated poor predictive performance with the Cancers dataset. RS-based DC and SVM-RFE-SVM performed robustly with all nine datasets. Compared with the nine reference models, RS-based DC received the least informative gene number (an average of 20.56), the highest average accuracy and the minimum standard deviation (9 %).

**Table 9:** Independent test accuracy and the number of informative genes (in parenthesis) among different models

Model	Leuk1	Lung1	Leuk2	SRBCT	Breast	Lung2	DLBCL	Cancers	GCM	Average
HC-TSP^a^	97.06	71.88	80.00	95.00	66.67	83.58	83.33	74.32	52.17	78.22 ± 13.97
	(4)	(4)	(4)	(6)	(8)	(8)	(10)	(20)	(26)	(10.00)
HC-K-TSP^a^	97.06	78.13	100	100	66.67	94.03	83.33	82.43	67.39	85.45 ± 13.12
	(36)	(20)	(24)	(30)	(24)	(28)	(46)	(128)	(134)	(52.22)
DT^a^	85.29	78.13	80.00	75.00	73.33	88.06	86.67	68.92	52.17	76.40 ± 11.13
	(2)	(4)	(2)	(3)	(4)	(5)	(5)	(10)	(18)	(5.89)
PAM^a^	97.06	78.13	93.33	95.00	93.33	100	90.00	87.84	56.52	87.91 ± 13.34
	(44)	(13)	(62)	(285)	(4822)	(614)	(3949)	(2008)	(1253)	(1450)
TSG^b^	97.06	81.25	100	100	86.67	95.52	93.33	79.73	67.39	88.99 ± 11.11
	(6)	(20)	(44)	(13)	(63)	(60)	(16)	(81)	(112)	(46.11)
mRMR-SVM	76.47	78.13	100	75.00	96.67	95.52	96.67	71.62	45.65	81.75 ± 17.54
	(7)	(13)	(19)	(9)	(97)	(120)	(16)	(89)	(57)	(47.44)
SVM-RFE-SVM	85.29	78.13	93.33	95.00	90.00	88.06	90.00	93.24	63.04	86.23 ± 10.08
	(5)	(9)	(8)	(3)	(7)	(9)	(13)	(29)	(199)	(31.33)
Entropy-based DC	91.18	78.13	86.67	100	83.33	88.06	93.33	78.38	47.83	82.99 ± 14.93
	(7)	(14)	(13)	(9)	(13)	(39)	(15)	(73)	(93)	(30.67)
*χ* ^2^-based DC	94.12	81.00	100	100	90.00	97.02	93.33	90.54	58.70	89.41 ± 12.91
	(23)	(18)	(30)	(31)	(33)	(42)	(23)	(95)	(90)	(42.78)
RS-based DC	94.12	84.38	100	100	93.33	98.51	90.00	90.54	71.74	91.40 ± 9.00
	(7)	(12)	(13)	(11)	(15)	(21)	(16)	(36)	(54)	(20.56)

^a^Results reported in [6], ^b^Results reported in [30]. The Average measurement was represented as the average value ± standard deviation. Bold values indicate the best prediction model of each dataset

The same modeling process was conducted for RS-based DC, Entropy-based DC and *χ*^2^-based DC to compare the merits of the defined score. As mentioned above, RS scores and *χ*^2^ scores utilize sample size information, whereas entropy scores only reflect the events ratio. Therefore, our RS-based DC and *χ*^2^-based DC have better predictive performance than Entropy-based DC method.

### Comparison of feature selection methods

An excellent feature selection method should perform well with various classifiers. We used four reference feature selection methods, mRMR, SVM-RFE, TSG and HC-K-TSP, to evaluate the performance of RS.

As shown in Table 10, with the informative genes selected by the five feature selection methods, the average independent prediction precisions of Naïve Bayes (NB) [31] and K-nearest neighbor (KNN) [32] on the nine datasets were clearly improved. However, surprisingly, the four reference feature selection methods were ineffective in the SVM classifier. This seems to challenge the conventional wisdom that feature selection should be effective in improving the performance of the model. Fortunately, RS still performed well with the SVM classifier upholding the conventional wisdom. For the SVM classifier, in three (Lung1, SRBCT and GCM) out of nine datasets, there was basically no improvement in performing feature selection, regardless of the feature selection technique. However, the NB and KNN classifiers did not always show such a phenomenon, possibly because SVM is not sensitive to feature dimensions; therefore, SVM could obtain very precise prediction without feature selection. RS was the only strategy that was better than no feature selection, on average, when combined with SVM, because on the Leuk1, Breast and Cancers datasets it showed a sufficiently large improvement was large enough, while it slightly reduced the precision of the prediction on the other datasets. Thus, the results indicated that RS is superior to the other four feature selection methods.

**Table 10:** Test accuracy of different classifiers with informative genes selected by different feature-selection methods

Classifier	Feature-selection method	Leuk1	Lung1	Leuk2	SRBCT	Breast	Lung2	DLBCL	Cancers	GCM	Average
NB	ALL^a^	85.29	81.25	100	60.00	66.67	88.06	86.67	79.73	52.17	77.76
	RS	94.12	84.38	100	85.00	93.33	88.06	90.00	85.14	71.74	87.97
	mRMR	79.41	68.75	100	90.00	93.33	97.01	96.67	70.27	45.65	82.34
	SVM-RFE	67.65	81.25	80.00	95.00	80.00	89.55	90.00	77.03	63.04	80.39
	TSG	91.18	84.38	93.33	100	86.67	94.03	100	71.62	65.22	87.38
	HC-K-TSP	91.18	81.25	100	80.00	80.00	95.52	86.67	77.03	65.22	84.10
KNN	ALL^a^	67.65	75.00	86.67	70.00^b^	63.33	88.06	93.33	64.86	34.78	71.71
	RS	97.06	78.13	93.33	90.00	93.33	95.52	93.33	72.97	43.48	84.13
	mRMR	70.59	68.75	80.00	80.00	96.67	86.57	100	54.05	36.96	74.84
	SVM-RFE	76.47	68.75	86.67	100	90.00	86.57	90.00	58.11	45.65	78.02
	TSG	91.18	75.00	93.33	100	80.00	88.06	96.67	74.32	39.13	81.97
	HC-K-TSP	88.24	87.50	86.67	85.00	83.33	94.03	93.33	64.86	52.17	81.68
SVM	ALL^a^	79.41	87.50	100	100	83.33	97.01	100	83.78	65.22	88.47
	RS	94.12	84.38	100	95.00	93.33	95.52	96.67	89.19	65.22	90.38
	mRMR	76.47	78.13	100	75.00	96.67	95.52	96.67	71.62	45.65	81.75
	SVM-RFE	85.29	78.13	93.33	95.00	90.00	88.06	90.00	93.24	63.04	86.23
	TSG	91.18	81.25	93.33	80.00	80.00	94.03	100	68.92	54.35	82.56
	HC-K-TSP	85.29	84.38	100	90.00	86.67	98.51	96.67	82.43	60.87	87.20

^a^Results reported in [6], ^b^The 30 reported in [3] is 70.00 after validation. Bold values indicate the best average accuracy in each classifier

### Comparison of generalization performance among different models

**Fig. 2:**
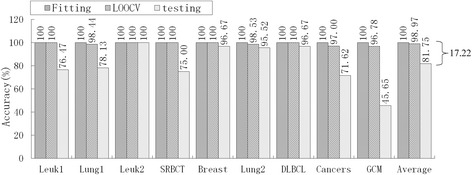
Accuracy of mRMR-SVM for fitting, LOOCV and independent test

**Fig. 3:**
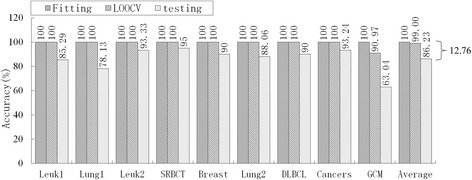
Accuracy of SVM-RFE-SVM for fitting, LOOCV and independent test

**Fig. 4:**
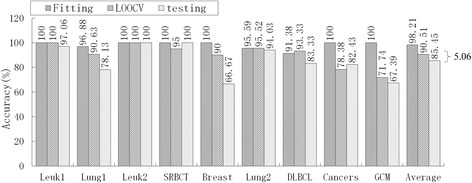
Accuracy of HC-K-TSP for fitting, LOOCV and independent test

**Fig. 5:**
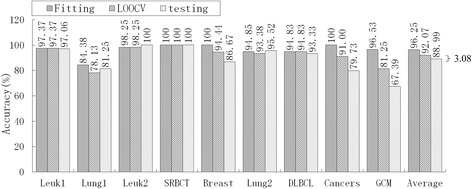
Accuracy of TSG for fitting, LOOCV and independent test

**Fig. 6:**
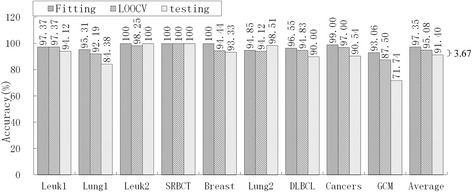
Accuracy of RS-based DC for fitting, LOOCV and independent test

Of the nine models in Table 9, PAM had an unacceptable informative gene number, DT had the lowest average accuracy (76.40 %), HC-TSP was similar to HC-K-TSP, and Entropy-based DC and *χ*^2^-based DC were similar to RS-based DC. Therefore, we selected five typical models, mRMR-SVM, SVM-RFE-SVM, HC-K-TSP, TSG and RS-based DC, for further evaluation of generalization performance by comparing the accuracy of fitting, LOOCV and independent testing. For LIBSVM[28], the LOOCV strategy was used to optimize penalty parameters C (C∈[2–5, 215]) and the gamma parameter γ(γ∈[2–15, 23]) in the kernel function. Suppose the training set has *n* samples, for a given combination of C and γ. We leave one as a validation sample and the other *n*-1 as sub-training samples, and acquire the LOOCV accuracy in this parameter combination after predicting n times. Traversing all parameter combinations, we acquire the highest LOOCV and the corresponding optimal C and γ. The optimal parameters and training set are used for constructing the predictive model. We apply this model to predict the training set and testing set, and obtain the fitting accuracy and independent testing accuracy, respectively. In sum, the fitting and LOOCV are the internal validation in this paper, and independent testing is the external validation. The results are shown in Fig. 2, 3, 4,5 and 6.

Obviously, over-fitting occurred with all five models; average accuracy always decreased monotonically from fitting through LOOCV to the independent test. For the mRMR-SVM and SVM-RFE-SVM models, which require parameter optimizations, the gaps between LOOCV average accuracy and test average accuracy were 17.22 % and 12.76 %, respectively. However, HC-K-TSP, TSG and RS-based DC models, which adopted a DC core and were parameter-free, tended to generate smaller gaps (5.06 %, 3.08 % and 3.67 %, respectively). For those models that required parameter optimizations, the test accuracy was always systematically less than the LOOCV accuracy for each dataset. For the DC core model, the test accuracy was even higher than LOOCV accuracy for some datasets, for example, the HC-K-TSP model for the SRBCT and Cancers datasets, TSG model for Lung1, Leuk2 and Lung2 datasets, and RS-based DC model for Leuk2 and Lung2 datasets.

Parameter optimizations may be responsible for SVM’s over-fitting? It could be argued that informative genes selected by mRMR and SVM-RFE are not the best feature subsets for mRMR-SVM and SVM-RFE-SVM models, respectively. RS resulted in better performance than the other four feature selection methods (Table 10). Therefore, we further compared the SVM performances with parameter optimizations or not, based on informative genes selected by RS. As shown in Table 11, parameter optimizations considerably improved the fitting and LOOCV accuracy of SVM. For the linear kernel and RBF kernel, the gaps between LOOCV average accuracy and test average accuracy with no parameter optimizations were 3.76 % and 1.90 %, respectively. However, the gaps with parameters optimization were 4.90 % and 9.43 %, respectively. That is, over-fitting is deepened by parameter optimizations in SVM.

**Table 11:** SVM performances with parameters optimization or not based on informative genes selected by RS

Parameters optimization	Kernel	Evaluation	Leuk1	Lung1	Leuk2	SRBCT	Breast	Lung2	DLBCL	Cancers	GCM	Average
No (fixed *C* = 1)	linear	Fitting	97.37	95.31	100	100	100	97.06	100	100	97.92	98.63
		LOOCV	97.37	81.25	98.25	98.41	94.44	94.12	96.55	96	77.78	92.69
		Testing	94.12	84.38	93.33	95	93.33	97.01	96.67	87.84	58.7	88.93
Yes	linear	Fitting	97.37	100	100	100	100	100	100	99	100	99.6
		LOOCV	97.37	90.63	100	100	98.15	94.12	100	96	81.25	95.28
		Testing	94.12	84.38	100	95	93.33	95.52	96.67	89.19	65.22	90.38
		*C*	0.25	32	0.03125	0.5	0.125	8	0.25	0.25	4	
No (fixed *C* = 1, γ = 1/*m*)	RBF	Fitting	97.37	87.50	100	100	100	91.18	100	88.00	45.14	89.91
		LOOCV	97.37	79.69	100	98.41	98.15	90.44	86.21	78.00	77.08	89.48
		Testing	94.12	78.13	100	95.00	93.33	97.01	93.33	85.14	52.17	87.58
Yes	RBF	Fitting	97.37	100	100	100	100	95.59	100	100	100	99.22
		LOOCV	97.37	90.63	100.00	98.00	98.15	94.12	100	98.00	82.64	95.43
		Testing	94.12	84.38	86.67	90.00	93.33	95.52	90.00	87.84	52.17	86.00
		*C*	8	2048	0.125	0.25	0.5	2	1	32768	32	
		γ	0.0125	0.0075125	0.25	0.125	0.25	0.0625	0.25	0.00390625	0.0625	

*C* is penalty parameters and *C*∈[2^−5^, 2^15^]; γ is gamma parameter in kernel function and γ∈[2^−15^, 2^3^]; *m* is features number of each SVM models

## Discussion

### Outlier adjustment and endpoint selection

A small number of outliers may affect gene ranking by changing the endpoints. Although not all gene expression values fit the normal distribution, the standard deviation of a normal distribution has good robustness for outlier adjustment when the probability of that distribution is unknown [33]. We compared independent test accuracies of RS-based DC with different significance level α (i. no adjustment, ii. α = 0.01, iii. α = 0.05). As shown in Table 12, the significance level α had an evident effect on classification performance, and 0.05 is the most appropriate choice for α. Endpoint selection is the nature of the binarization procedure for the vertical comparison of gene evaluation. TSG uses the mean of gene expression values as its endpoint [9]. In this paper, the endpoint defined by formula (11) is based on Fisher’s discriminant principle. We also compared independent test accuracies of RS-based DC with different endpoint selection approaches. As shown in Table 12, the endpoint selection approach has very little influence on classification performance.

**Table 12:** Independent test accuracy of RS-based DC with different outlier adjustment and endpoint selection approach

*α*	*EP* selection	Leuk1	Lung1	Leuk2	SRBCT	Breast	Lung2	DLBCL	Cancers	GCM	Average
No adjustment	Formula (11)	94.12	84.38	93.33	95.00	90.00	100.00	90.00	81.08	60.87	87.64
0.01	Formula (11)	94.12	84.38	93.33	100	90.00	97.02	90.00	90.54	63.04	89.16
0.05	Formula (11)	94.12	84.38	100	100	93.33	98.51	90.00	90.54	71.74	91.40
0.05	Mean	94.12	84.38	100	100	93.33	97.01	90.00	90.54	71.74	91.24

### Entropy and complexity

In this study, a novel score measure, RS, is proposed based on complexity. Complexity and entropy are very similar. The former takes sample size information into account in addition to entropy. As scores are calculated based on percentages, sample size information is not fully utilized in the latter. For example, suppose three white balls and seven black balls are in a system, the entropy (*H*) is 0.88. In another case, suppose all the counts are multiplied by 10, *i.e*. 30 white balls and 70 black balls; *H* is identical to the previous case. The additional information related to the additional sample size is completely ignored in entropy measures. For Entropy-based DC, we used entropy in place of the complexity used in RS-based DC. The results are shown in Table 9. The same modeling process was conducted for the two models, but Entropy-based DC had poorer predictive performance than RS-based DC. This result shows that the additional information associated with sample size can improve a model’s predictive performance.

### Horizontal and vertical evaluation of gene pairs

Background differences between pair-wise genes and among samples are fairly common in microarray expression data, and result in very diverse joint effect patterns. It is difficult to fairly evaluate all of the patterns with a single-strategy. As shown in Table 13, a vertical comparison cannot highlight gene G_1141_ and G_4940_ in the GCM dataset, and a horizontal comparison cannot highlight gene G_6678_ and G_3330_ in the Lung1 dataset. RS, however, highlighted the two pairs of genes by integrating vertical comparison with horizontal comparison.

**Table 13:** Horizontal and vertical comparison of gene pairs in real data

GCM dataset	Horizontal comparison		Vertical comparison			
	*X*_1141_ > *X*_4940_	*X*_1141_ < *X*_4940_	*X*_1141_ > 33 & *X*_4940_ > 232	*X*_1141_ > 33 & *X*_4940_ < 232	*X*_1141_ < 33 & *X*_4940_ > 232	*X*_1141_ < 33 & *X*_4940_ > 232
Class 11	2	9	3	1	1	6
Class 12	10	1	0	0	0	11
*p-value*	0.0027		0.0908			
Lung1 dataset	Horizontal comparison		Vertical comparison			
	*X*_6678_ > *X*_3330_	*X*_6678_ < *X*_3330_	*X*_6678_ > 7439 & *X*_3330_ > 335	*X*_6678_ > 7439 & *X*_3330_ < 335	*X*_6678_ < 7439 & *X*_3330_ > 335	*X*_6678_ < 7439 & *X*_3330_ < 335
Class 1	41	3	18	11	10	5
Non-Class 1	19	1	0	2	1	17
*p-value*	0.7806		2.0716 × 10^−7^			

### Direct classifier

Parameters need to be optimized and adjusted, *e.g*. the parameters of a kernel function in SVM, and the connection weights of neurons in an artificial neural network. This is the primary reason for classifier over-fitting. SVM integrates the minimum structure risk and the maximal margin and transduction inference, and thereby should be able to efficiently control over-fitting. SVM-RFE-SVM and mRMR-SVM have the highest LOOCV accuracies of those SVM classifiers we tested, 99 % and 98.97 %, respectively. Therefore, these two SVM variants should theoretically both receive high test accuracy. However, results were not as good as expected; obvious over-fitting still appeared (See Fig. 2 and Fig. 3) and deepened by parameter optimizations (See Table 11).

HC-K-TSP, TSG and RS-based DC models, on the other hand, simultaneously received high LOOCV accuracy, high independent test accuracy, and a small gap. Test accuracy higher than LOOCV accuracy appeared in different datasets for the three models, excluding the possibility that DC preferred a specific dataset. The three models have different defined scores and different feature selection methods, only having the same DC core; therefore, we believe that DC plays an important role in effectively controlling over-fitting.

### Paired votes based on binary-discriminative informative genes

In most cases, an informative gene can distinguish between just a few classes much more robustly than all of the classes in a multi-class dataset. Therefore, it is necessary to transform datasets from multi-class to binary-class with a “one versus one” (OVO) or an OVR approach. For an *m*-class dataset, OVO gets incredibly complicated, especially with a big *m*, as the OVO has to build *m*(*m*-1)/2 binary-classifiers. OVR only needs to build *m* binary-classifiers; however, a serious unbalance between the number of positive samples and negative samples may distort prediction resulting in non-unique calls. Therefore, we employ paired votes based on binary-discriminative informative genes that integrate OVO with OVR. We first build *m* binary-classifiers with OVR to select *m* BDIG subsets, then build *m*-1 binary-classifiers with OVO to perform paired votes. For each paired votes between Class_*t*_ and Class_*w*_, feature subset {BDIG_Class*t*_ ∪ BDIG_Class*w*_} was binary-discriminative and the sample sizes were balanced. Paired votes based on binary-discriminative informative genes only built 2 *m*-1 binary-classifiers and received robust prediction precision.

### Biological relevance of informative genes selected by *RS*

Do informative genes selected by *RS* have any biological relevance for a particular tissue/cancer type? This is particularly relevant considering that even a random set of genes may be a good predictor for defining cancer samples [34]. In our study we scanned these potentially informative genes against PubMed. Two examples illustrate: for the Leuk2 dataset, 13 genes out of 12,582 were selected as informative genes by our method, of which ten genes are reported in PubMed as being related to tumors, and seven genes are reported as being related to leukemia (see Table 14). For the Cancers dataset (prostate, breast, lung, ovary, colorectum, kidney, liver, pancreas, bladder/ureter, and gastroesophagus), 36 genes out of 12,533 were selected as informative genes, of which 34 genes are reported related to be tumor related in PubMed (see Table 15). Clearly, most of informative genes selected by RS are supported by PubMed references (Informative genes selected by RS method of nine datasets see Additional file 1).

**Table 14:** The 10 tumor related genes selected by RS on original training group of Leuk2 dataset

Symbol	Synonym(s)	Entrez Gene Name	Related carcinoma and Ref.
FTL	LFTD, NBIA3	ferritin, light polypeptide	breast cancer [35]
PDK1		pyruvate dehydrogenase kinase, isozyme 1	leukemia [36]
POU2AF1	BOB1, OBF-1, OBF1, OCAB	POU class 2 associating factor 1	leukemia [37]
KLRK1	CD314, D12S2489E, KLR, NKG2-D, NKG2D	killer cell lectin-like receptor subfamily K, member 1	leukemia [38]
KCNH2	ERG-1, ERG1, H-ERG, HERG, HERG1, Kv11.1, LQT2, SQT1	potassium channel, voltage gated eag related subfamily H, member 2	leukemia [39]
VLDLR	CAMRQ1, CARMQ1, CHRMQ1CH, VLDLR	very low density lipoprotein receptor	breast cancer [40]
MEIS1		Meis homeobox 1	leukemia [41]
MLXIP	MIR, MONDOA, bHLHe36	MLX interacting protein	leukemia [42]
NF2	ACN, BANF, SCH	neurofibromin 2 (merlin)	tumor suppressor [43]
MAP3K5	ASK1, MAPKKK5, MEKK5	mitogen-activated protein kinase kinase kinase 5	leukemia [44]

**Table 15:** The 34 tumor related genes selected by RS on original training group of Cancers dataset

Symbol	Synonym(s)	Entrez Gene Name	Related carcinoma and Ref.
CYP1A1	AHH, AHRR, CP11, CYP1, P1-450, P450-C, P450DX	cytochrome P450, family 1, subfamily A, polypeptide 1	lung cancer [45]
PTPRZ1	HPTPZ, HPTPzeta, PTP-ZETA, PTP18, PTPRZ, PTPZ, R-PTP-zeta-2, RPTPB, RPTPbeta, phosphacan	protein tyrosine phosphatase, receptor-type, Z polypeptide 1	lung cancer [46]
WT1	AWT1, EWS-WT1, GUD, NPHS4,WAGR, WIT-2, WT33	Wilms tumor 1	leukemic [47]
ANGPT2	AGPT2, ANG2	angiopoietin 2	lung cancer [48]
LGALS1	GAL1, GBP	lectin, galactoside-binding, soluble, 1	hepatocellular carcinoma [49]
ACPP	5'-NT, ACP-3, ACP3	acid phosphatase, prostate	prostate cancer [50]
GC	DBP, DBP/GC, GRD3, HEL-S-51, VDBG, VDBP	group-specific component (vitamin D binding protein)	bladder cancer [51]
PRMT1	ANM1, HCP1,HRMT1L2, IR1B4	protein arginine methyltransferase 1	breast cancer [52]
NOX1	GP91-2, MOX1, NOH-1, NOH1	NADPH oxidase 1	colon cancer [53]
ADH7	ADH4	alcohol dehydrogenase 7 (class IV), mu or sigma polypeptide	gastric cancer [54]
DSG3	CDHF6, PVA	desmoglein 3	bladder carcinoma [55]
NKX2-1	BCH, BHC, NK-2, NKX2.1, NKX2A, T/EBP, TEBP, TITF1, TTF-1, TTF1	NK2 homeobox 1	lung cancer [56]
EFHD1	MST133, MSTP133, PP3051, SWS2	EF-hand domain family, member D1	colorectal cancer [57]
EREG	EPR, ER, Ep	epiregulin	colorectal cancer [58]
DHRS2	HEP27, SDR25C1	dehydrogenase/reductase (SDR family) member 2	breast cancer [59]
ENPEP	APA, CD249, gp160	glutamyl aminopeptidase (aminopeptidase A)	prostate cancer [60]
SCGB2A2	MGB1, UGB2	secretoglobin, family 2A, member 2	breast cancer [61]
KRT13	CK13, K13, WSN2	keratin 13, type I	breast cancer [62]
SERPINC1	AT3, AT3D, ATIII, THPH7	serpin peptidase inhibitor, clade C (antithrombin), member 1	bladder cancer [63]
SLC12A2	BSC, BSC2, NKCC1, PPP1R141	solute carrier family 12 (sodium/ potassium/chloride transporter),member 2	esophageal squamous cell carcinoma [64]
IRF4	LSIRF, MUM1, NF-EM5, SHEP8	interferon regulatory factor 4	hematological malignancies [65]
GPA33	A33	glycoprotein A33 (transmembrane)	colorectal cancer [66]
BCAT1	BCATC, BCT1, ECA39, MECA39, PNAS121, PP18	branched chain amino-acid transaminase 1, cytosolic	colorectal cancer [67]
COL10A1		collagen, type X, alpha 1	breast cancer [68]
CEL	BAL, BSDL, BSSLL, CEase, FAP, FAPP, LIPA, MODY8, CEL	carboxyl ester lipase	pancreatic cysts [69]
NPC2	EDDM1, HE1	Niemann-Pick disease, type C2	liver cancer [70]
CDH17	CDH16, HPT-1, HPT1	cadherin 17, LI cadherin (liver-intestine)	gastric cancer [71]
MEIS1		Meis homeobox 1	pancreatic cancer [72]
KLK3	APS, KLK2A1, PSA, hK3	kallikrein-related peptidase 3	prostrate [73]
CXCL13	ANGIE, ANGIE2, BCA-1, BCA1, BLC, BLR1L, SCYB13	chemokine (C-X-C motif) ligand 13	breast cancer [74]
ELA3A	ELA3,ELA3A	chymotrypsin-like elastase family, member 3A	pancreatic carcinoma [75]
IRX5	HMMS, IRX-2a, IRXB2	iroquois homeobox 5	prostate cancer [76]
VCAM1	CD106, INCAM-100	vascular cell adhesion molecule 1	ovarian cancer [77]
P4HB	CLCRP1, DSI, ERBA2L, GIT, P4Hbeta, PDI, PDIA1, PHDB, PO4DB, PO4HB, PROHB	prolyl 4-hydroxylase, beta polypeptide	Glioblastoma multiforme [78]

## Conclusion

Gene selection and classifier choice are two key issues in the analysis of tumor microarray expression data. Gene selection depends on an evaluation strategy and on a defined score. Diverse patterns of gene pairs can be highlighted more fully by integrating a vertical comparison with a horizontal comparison strategy. The RS score and the *χ*^2^ score, which both consider events ratios as well as events frequencies, were superior to Δ_*ij*_ scores and entropy scores. Parameter optimizations are the main reason for over-fitting classifiers, a DC core classifier can effectively control over-fitting. RS-based DC (Source code of RS-based DC see Additional file 2), which takes into account all of the above factors, receives the highest average independent test accuracy, the smallest informative average gene number, and the best generalization performance. This was confirmed by testing our method on nine bench-mark multi-class gene expression datasets, compared with the nine reference models and the four reference feature selection methods.

## Additional files

Additional file 1:
**The binary-discriminative informative genes selected by RS method of nine datasets.** (XLS 26 kb) Additional file 2:
**Source code of RS-based DC.** (RAR 768 kb)

## Abbreviations

TSP: Top scoring pair
TSG: Top score genes
RS: Relative simplicity
RS-based DC: relative simplicity-based direct classifier
LOOCV: Leave-one-out cross validation
OVR: One versus rest
C: Complexity
IRS: Integrated RS score
MCC: Matthew correlation coefficient
BDIG: Binary-discriminative informative genes. K-TSP, k top scoring pairs
HC-TSP: Multi-class extension of TSP with hierarchical classification scheme
HC-k-TSP: Multi-class extension of k-TSP with hierarchical classification scheme
PAM: Prediction Analysis of Microarray
SVM: Support Vector Machine classification
NB: Naive bayes
KNN: K-nearest neighbor
OVO: One versus one
